# Biological Function and Molecular Mapping of M Antigen in Yeast Phase of *Histoplasma capsulatum*

**DOI:** 10.1371/journal.pone.0003449

**Published:** 2008-10-17

**Authors:** Allan Jefferson Guimarães, Andrew John Hamilton, Herbert Leonel de M. Guedes, Joshua Daniel Nosanchuk, Rosely Maria Zancopé-Oliveira

**Affiliations:** 1 Division of Infectious Diseases, Department of Medicine and Microbiology and Immunology, Albert Einstein College of Medicine of Yeshiva University, Bronx, New York, United States of America; 2 Laboratório de Micologia - Setor de Imunodiagnóstico - Instituto de Pesquisa Clínica Evandro Chagas, Fundação Oswaldo Cruz, Rio de Janeiro, Brazil; 3 St John's Institute of Dermatology, Guy's Hospital, King's College, London, United Kingdom; 4 Laboratório de Bioquímica de Proteínas e Peptídeos, Instituto Oswaldo Cruz, Fundação Oswaldo Cruz, Rio de Janeiro, Brazil; University of Minnesota, United States of America

## Abstract

Histoplasmosis, due to the intracellular fungus *Histoplasma capsulatum*, can be diagnosed by demonstrating the presence of antibodies specific to the immunodominant M antigen. However, the role of this protein in the pathogenesis of histoplasmosis has not been elucidated. We sought to structurally and immunologically characterize the protein, determine yeast cell surface expression, and confirm catalase activity. A 3D-rendering of the M antigen by homology modeling revealed that the structures and domains closely resemble characterized fungal catalases. We generated monoclonal antibodies (mAbs) to the protein and determined that the M antigen is present on the yeast cell surface and in cell wall/cell membrane preparations. Similarly, we found that the majority of catalase activity was in extracts containing fungal surface antigens and that the M antigen is not significantly secreted by live yeast cells. The mAbs also identified unique epitopes on the M antigen. The localization of the M antigen to the cell surface of *H. capsulatum* yeast and the characterization of the protein's major epitopes have important implications since it demonstrates that although the protein may participate in protecting the fungus against oxidative stress it is also accessible to host immune cells and antibody.

## Introduction

The dimorphic fungus *Histoplasma capsulatum* is the causative agent of the systemic mycosis histoplasmosis. *H. capsulatum* has a worldwide distribution with areas of high endemicity, such as the Mississippi and Ohio river valleys of the USA and the South and Southeast of Brazil [1]. Human infection primarily occurs after inhalation of microconidia, which are deposited in the distal alveoli where they are phagocytosed by macrophages and undergo morphogenic change into a yeast form [2], [3]. In the phagosome of these cells, the fungus is exposed to stress conditions including changes in pH and reactive oxygen species [4]. One mechanism that *H. capsulatum* purportedly utilizes to evade oxidative stress is the production of catalases [5]. Catalases are ubiquitous enzymes that, independent of their origin, function to degrade two molecules of hydrogen peroxide into two of water and one of oxygen.

Catalase species are widely distributed in the prokaryotic kingdom and lower eukaryotes (catalase-peroxidase), as well as in higher eukaryotes (homotetramic, heme containing enzymes) [6], [7]. Cellular metabolism usually generates toxic species mediated by products from the univalent reduction of molecular oxygen, including species such as superoxide radicals (O_2_^−^), peroxide (O_2_^−2^) and hydroxyl (OH^−^). Additionally, infectious organisms can be damaged by exposure to these same radicals. Diverse mechanisms have been described to damage as a consequence of oxidative stress on microbes, including peroxisome proliferation and DNA breakage [5], [8].

Microbial enzymes, such as catalases, involved in defense against oxidative stress have been associated with pathogenicity and virulence in certain human fungal pathogens. *Aspergillus fumigatus* has two mycelial catalases and one conidial catalase whose actions counteract the oxidative defense reaction mechanism in host phagocytes [9]. Induction of catalases in *Paracoccidioides brasiliensis* protects the fungus against endogenously produced oxygen radicals and H_2_O_2_ [10]. *P. brasiliensis* catalases are transcriptionally up-regulated during yeast development and also increase during mycelium to yeast conversion. Although rarely pathogenic, *Saccharomyces cerevisae* has two catalases, one peroxisomal catalase (catalase A) and one cytosolic catalase (catalase T), which play especially important roles in tolerance to oxidative stress in the adaptive response of these cells [11]. Catalase disruptants of *Candida albicans* are fully viable under normal culture conditions, but are extremely sensitive to oxidative stress by hydrogen peroxide and are cleared more rapidly than wild type cells in a murine infection model [12]. Although the role of catalase in *Penicillium marneffei* has not been elucidated, its catalase gene displays a high level of expression when temperature is shifted from 25°C to 37°C inducing the morphogenesis of the fungus from a mould to yeast forms. The up-regulation of the *P. marneffei* catalase purportedly promotes the survival of this dimorphic fungus in host cells [13]. A catalase (catalase B) from *Magnaporthe grisea* is important for the maintenance of fungal cell-wall integrity during plant cell infection and invasion [14]. The catalase B gene is 600-fold up-regulated during infection and disruption of the gene attenuates the virulence of the fungi.

Three different catalases have been described in *H. capsulatum* and each catalase gene is present in a single copy [5]. Catalase P is a small-subunit monofunctional peroxisomal catalase, composed of a single chain of 57 KDa that has high similarity with the monofunctional catalase P of *P. brasiliensis* [10]. *H. capsulatum* catalase A and B are large-subunit bifunctional (catalase-peroxidase) enzymes that usually form quaternary structures in solution [6], [7]. Catalase B and P are constitutively expressed, whereas catalase A is induced upon H_2_O_2_ stress. Catalase B is also known as the M antigen, which is a major diagnostic antigen of *H. capsulatum* as it elicits both humoral and cellular immune responses [1], [15]–[19]. It is noteworthy that the M antigen (catalase B) has previously been described as a secreted enzyme [15], [20], [21].

The M glycoprotein induces the first precipitins to arise in acute histoplasmosis and is also present in all subsequent phases of disease [22], [23]. The immunodominant M antigen of *H. capsulatum* has previously been purified using chromatographic methods and characterized by a combination of immunochemical assays. The relative molecular weight varies depending on glycosylation and ranges from ∼70–94 kDa, and the molecule contains species-specific and non-specific protein and carbohydrate epitopes [24]–[26]. Cross-reactivity with sera from patients with infections other than histoplasmosis has been attributed to the presence of these glycosylated residues and can be minimized by chemical and enzymatic deglycosylation methods [22], [23], [26], [27]. Furthermore, the gene sequence of the M antigen has allowed for the design of primers to develop a highly specific and sensitive PCR for the identification of *H. capsulatum* atypical isolates [28], suggesting that the M antigen has specific epitopes, as well as common epitopes shared with numerous other catalases from diverse sources.

The purpose of our study is to show that the M glycoprotein is expressed on the fungal cell surface where it may interact with host cells and antibody. We demonstrate that the most immunogenic epitopes are located on the surface of the proposed 3-D model and are thus sterically accessible to antibodies and effector cells of the immune system. Also, we identified a species-specific mAbs that may be applied to the serodiagnosis of histoplasmosis.

## Methods

### Fungal strains and culture conditions

*H. capsulatum* strain ATCC G217B and CDC6623 (ATCC 26320) were obtained from the American Type Culture Collection (ATCC, Rockville, Maryland, USA). Yeast forms were maintained by cultivation at 37°C in Ham's F-12 medium as previously described [29]. *P. brasiliensis* strain CIB Pb 339 was obtained from the Corporación para Investigaciones Biológicas (CIB, Colombia) and yeasts were cultivated in Fava Netto's medium at 37°C for 6 days [30], while filamentous forms were cultivated for 15 days at 25°C in the same medium. *Sporothrix schenckii* (Ssl7 - St. John's Institute of Dermatology, London, United Kingdom) yeasts were grown in Brain Heart Infusion (BHI) broth for 3 days at 37°C [31]. *Blastomyces dermatitidis* (NCPF 4076-National Collection of Pathogenic Fungi, Colindale, London, United Kingdom) yeasts were grown in BHI at 37°C for five days [32]. *A. fumigatus* (strain 3111 – Laboratório de Micologia, Instituto de Pesquisa Clínica Evandro Chagas, FIOCRUZ, Brazil) was grown in Sabouraud Broth at 37°C for 96 h (cellular extract) or 3 weeks (culture filtrate). All the strains were incubated with shaking at 150 rpm. *Mycobacterium tuberculosis* cell extract was a gift from Dr. Beatriz Gomez (CDC, Atlanta, GA).

### Antigen preparations

Antigens were obtained from *H. capsulatum* yeast and other microorganisms that often cross-react with *H. capsulatum* by immunoprecipitation techniques [1], [33], [34]. Yeast cells of *H. capsulatum*, *P. brasiliensis*, *S. schenckii*, and *B. dermatitidis* were harvested by centrifugation and cytoplasmic yeast antigens (CYA) were prepared as described by Hamilton *et al* [35], [36]. CYA antigens were obtained by suspending 10^9^ yeast cells in phosphate buffered saline (PBS- 10 mM NaH_2_PO4, 10 mM Na_2_HPO_4_ pH 7.0 and 150 mM NaCl) containing protease inhibitor cocktail (Roche IN, USA) followed by disruption of the cells using a bead beater at 4°C with 10 cycles of 1 minute followed by 1 minute incubations on ice. The lysates were then centrifuged at 11,300×g for 20 min and the supernatant containing the cytoplasmic fraction were collected. Filtered antigens from *A. fumigatus* and *P. brasiliensis* filamentous cultures were prepared by filtration of the culture supernatant using a 0.45 µm Millipore membrane (Billerica, MA USA) and the flow through concentrated 100×. Two other antigenic preparations from *A. fumigatus* and *M. tuberculosis* were obtained by disruption of the cells using a bead beater (Biospec Products, Bartlesville, Okla.) at 4°C with 10 cycles of 1 min alternating with incubations on ice for 1 min [37]. Purified histoplasmin was prepared from mycelial cultures of *H. capsulatum* CDC6623 (ATCC 26320) as described [24]. Histoplasmin fractions were pooled and glycosyl residues oxidized by treatment with sodium *m*-periodate (NaIO_4_) [26]. Protein concentrations were determined using a dye-binding assay as described [38], using bovine serum albumin as a standard.

### Sequence analysis and phylogeny of the M antigen

Studies of the primary sequence of the M antigen were done based on the sequence of accession number AAB84182 from Swiss-Prot/ TrEMBL. JPRED (www.compbio.dundee.ac.uk/www-jpred/) was used to determine sequences similar to the M antigen and predict the secondary structure. Catalase from *Penicillium vitale* (PDB ID 2IUF-E, 1.71-Å resolution) [39] and *Escherichia coli* (PDB ID 1QF7-A 2.20-Å resolution) [40] were used as templates in the modeling procedure. To study the phylogenetic relationship of M antigen among catalases of the *Ascomycota* class and determine the phylogenetic distance of the two catalases used to construct the M antigen model, we identified similar sequences using the BLAST algorithm (http://www.ncbi.nlm.nih.gov/blast) and sequences databases (GenBank and Swiss-Prot/ TrEMBL). Alignment of the identified sequences was done using the Clustal W and Meg Align (5.03) software from DNASTAR package (Madison, Wisconsin University, USA) and homologous proteins to this antigen were determined. This resulted in a final alignment of 26 taxa and 901 sites using MEGA program version 4 [41]. The neighbor-joining with maximum-parsimony method was used to construct the phylogenetic tree. All bootstrap support values were based on 500 replicates.

### Homology modeling

The sequence of the M antigen was submitted to the Swiss-model automated modeling server [42] to derive a 3D model using the two catalases cited above as a template. The study was assessed with the analytical tools available in SwissPDB viewer v. 3.6 and with the programs in the PROCHECK suite for model validation [43]. The molecular model of M antigen was submitted to model refinement and energy minimization conducted with the GROMOS96 v.43B1 force field implemented in the SwissPDB viewer v.3.7b2. The final refined theoretical structure of M antigen was achieved by a harmonic constraint with 20 steps of steepest descent in all residues followed by 1,000 steps of steepest descent and conjugated gradient minimization methods until the energy difference between the two steps was below 0.01 kJ/mol. Additional refinements of the M antigen model were performed using a similar minimization protocol in which the type of residues constrained during the minimization were varied: initially, only residues out of the most favored regions of the Ramachandran plot were allowed to move, then residues with a high model B-factor and/or force field energy were included.

### Cloning, expression and purification of the recombinant M antigen

PCR was used to amplify the sequence of the M antigen from 50 ng of cDNA as a template in the presence of 2 U of Platinum *Taq* DNA polymerase (Invitrogen, CA, USA), 50 mM KCl, 10 mM Tris-HCl (pH 8.4), 2.5 mM MgCl_2_, 100 µM of each deoxynucleotide triphosphate, 0.5 µM sense primer cDNAF (5′- CGCAATTCAGATCTGACCCTACGGACCAG-3′) and anti-sense primer cDNAR (5′-ACCAAGCTTCTAGCTTCTATCCAACGGGAA-3′). The cDNAF primer contained the restriction enzyme site for BglII and the cDNAR primer contained HindIII. The conditions of the PCR were 94°C for 2 min, thirty-five cycles 94°C for 45 s, 55°C for 45 s and 72°C for 1 min and a final extension for 2 min at 72°C. The PCR product was digested with BglII and HindIII and ligated into pQE40 expression plasmid (Qiagen, CA, USA). The construct was used to transform M15 *E. coli* strains (Qiagen, CA, USA) and the bacteria were plated on medium contained ampicillin (100 µg/mL) and kanamycin (25 µg/mL). Clones were screened by PCR using the same pair of primers described above and by restriction mapping of the plasmid, followed by sequencing. One positive clone was selected (M-5) and grown at 37°C with shaking in liquid Luria-Bertani medium containing selective antibiotics. Culture was performed until the absorbance reached 0.4–0.6 at 600 nm and recombinant protein expression was induced by addition of IPTG (0.1 mM). Cells were incubated at 37°C for 4 h with 300 rpm shaking. Cells were harvested at 1,100×*g* and suspended in a buffer containing 5 mM imidazole, 20 mM Tris (pH 7.9), 500 mM NaCl and the protease inhibitor cocktail (Roche, Mannheim, Germany). Cells were disrupted using glass beads and ultrasonication with 10 cycles of 1 min followed by incubations on ice. The cell lysate was centrifuged at 11,300×*g* to separate the soluble from the insoluble particles. The insoluble pellet was suspended in a buffer containing 6 M urea, 5 mM imidazole, 20 mM Tris (pH 7.9), 500 mM NaCl and the insoluble fractions were separated by centrifugation at 11,300×*g*. Supernatant was passed through a 0.22 µm PTFE syringe filter (Fisher Scientific, USA) and loaded onto a pre-equilibrated Ni-NTA agarose column (Quiagen CA, USA). The column was washed with 10 bed volumes of washing buffer (10 mM imidazole, 6 M urea, 20 mM Tris pH 7.9, 500 mM NaCl) and the recombinant M antigen was eluted with 500 mM imidazole, 6 M urea, 20 mM Tris pH 7.9, 500 mM NaCl. The eluted fractions were measured in a spectrophotometer at 280 nm and the fractions containing the higher amount of proteins were evaluated by SDS-PAGE for the presence of the recombinant M protein (rM). Fractions containing the rM antigen were pooled for use in subsequent experiments. For immunizations, rM protein was dialyzed against a buffer containing 10 mM Tris pH 7.3 and 150 mM NaCl with decreasing urea concentrations.

### Epitope mapping of the rM antigen

In silico analysis was performed to determine sequences displaying antigenic properties. Epitope mapping predictions for antigenicity were calculated using the Jamenson-Wolf algorithm in the Protean program (DNASTAR Inc, Madison, Wis., USA). Protean program and ProtoParame ProtScale (www.expasy.ch) were used to characterize the physical-chemical properties of the identified antigenic regions. The strategy used for epitope mapping of the rM was the fragmentation of the M antigen in three non-overlapping fragments, F1 (from amino acid 18 to 211), F2 (from 212 to 442), and F3 (from 443 to 705). The fragments were obtained by PCR reaction using a cDNA template and the primers designed specifically for the amplification of each fragment are shown in the Table 1. The PCR, cloning strategy and purifications were performed as described above for the entire protein. The yield of purification and the presence of the fragments of interest were assessed by SDS-PAGE and the proteins were dialyzed to remove urea. The protein concentration was obtained by a dye-binding assay and immunoblot was used to evaluate the binding of the panel of mAbs produced.

**Table 1:** PCR primers used to generate the fragments of the rM antigen.

Fragments	“primers”	Sequences
F1	CDNAF	**5′-CGCAATTCAGATCTGACCCTACGGACCAG-3′**
	F1R	**5′-CATAAGCTTTGCCCAGAAGAGGGCATGCAA-3′**
F2	F2F	**5′GCAGAT TATGTCAGGACATGGAATCCCTC 3′**
	F2R	**5′-CATAAGCTTACGCCCAGGTGCGGTGAAGAA T-3′**
F3	F3F	**5′-CGTAGATCTATGGTAAATGGACCACTAGTG C-3′**
	CDNAR	**5′-ACCAAGCTTCTAGCTTCTATCCAACGGGAA-3′**

Fragments of the rM antigen were used to determine the overall antibody response in sera of patients with histoplasmosis and to evaluate the relative reactivity of each fragment. Sera were obtained from fourteen patients with culture confirmed histoplasmosis from the Instituto de Pesquisa Clínica Evandro Chagas (FIOCRUZ, Rio de Janeiro, RJ, Brazil). Additionally, the patients had positive immunodiffusion results to the M antigen using histoplasmin. The serum samples were from a serum bank at FIOCRUZ with approved use by the Ethics Committee of the institution and were stored at −20°C prior to use. The reactivity for *H. capsulatum* of each patient's serum was measured by ELISA as described [33]. Briefly, rM antigen and fragments were diluted in coating buffer (63 mM; pH 9.6) to a concentration of 0.01 µg/µL and 50 µL/well were added to a 96-well microtiter plates (Nunc-Immuno Starwell, MaxiSorp Surface) and incubated for 1 h at 37°C followed by an overnight incubation at 4°C. Plates were washed three times with washing buffer (TBS) and blocked with 200 µl blocking buffer (1% BSA diluted in TBS) for 2 h at 37°C. Then the plates were again washed three times with washing buffer and serum samples were added in duplicate at a dilution of 1∶1,000 in 50 µL of blocking buffer. Plates were incubated for 1 h at 37°C and after three further washes, plates were incubated with either anti-human IgM, IgA, IgG or IgE alkaline phosphatase conjugate (Jackson Immunoresearch) 1∶2000 diluted in 100 µL of blocking buffer, at 37°C for 1 h. After three subsequent washes, the reaction was developed using 50 µL of pNPP diluted in substrate buffer (1 mM MgCl_2_, 50 mM Na_2_CO_3_) and measured at 405 nm. The relative reactivity of the sera to each fragment was calculated based on the value of the absorbance obtained for each sample for a specific fragment, divided by the sum of all the absorbances.

### Generation of monoclonal antibodies (mAbs) to rM

Four 6-week old female BALB/c mice were bled and the sera tested to document the absence of antibodies to rM antigen. The animals were then immunized with intraperitoneal injections of 20 µg of the rM antigen emulsified 1∶1 (vol/vol) in complete Freund's adjuvant (Sigma). At 2 and 4 weeks after the first immunization, additional doses of 20 µg of rM protein emulsified in incomplete Freund's adjuvant were administered. Two weeks after each immunization, sera were obtained and analyzed for reactivity to rM assessed by an indirect ELISA developed for this study using 1 µg of rM antigen diluting in 50 µL (1 µg/well) of coating buffer (63 mM Na_2_CO_3_, 63 mM NaHCO_3_ pH 9.6) to coat each well of the 96-well plates. After 3 washes with TBS (10 mM Tris-HCl pH7.2, 150 mM NaCl, 0.1% Tween 20), plates were blocked with 1% bovine serum albumin (BSA) in TBS buffer for 1 h at 37°C. Animal serum was diluted 1∶100 in 1% BSA blocking buffer and added to the wells in duplicate. Serial dilutions were made and the plates were incubated for 1 h at 37°C. After three washes, plates were further incubated with a 1∶2,000 dilution of anti-mouse immunoglobulin (Ig) alkaline phosphatase conjugate (Jackson Immunoresearch) for 1 h at 37°C, and then the reactions were developed with p-nitrophenyl phosphate (pNPP) and read at 405 nm. The animal with the highest titer two weeks after the last immunization (week 6) was boosted intravenously with 20 µg of antigen for 3 consecutive days and then its splenocytes were used to produce hybridomas according to protocol [36]. Isotyping of mAbs was assessed using the Isotyping Kit Enzyme Immunoassay (Boehring Mannhein, Germany) according to the manufacturer's instructions. The concentration of mAbs was determined by capture ELISA as described [44]. Briefly, ELISA plates were coated with unlabeled antibodies specific to different Ig isotype and then blocked with 1% BSA in TBS. The purified antibodies to rM protein and Ig standards were serially diluted and incubated for 1 h at 37°C. A secondary isotype specific mAb conjugated with alkaline phosphatase was added and the reaction developed using p-nitrophenyl phosphate (pNPP). The absorbances of the samples were compared to the Ig standard and the concentration determined.

### Evaluation of the mAb specificity

A membrane based technique was used to evaluate the reactivity of the generated mAbs to rM antigen, *H. capsulatum* CYA, purified and metaperiodate treated histoplasmin (ptHMIN), *P. brasiliensis* CYA, *P. brasiliensis* filtered supernatant- Pb(f), *A. fumigatus* filtered supernatant- Af(f), sonicated cells of *A. fumigatus* and *M. tuberculosis*, *S. schenckii* CYA, *B. dermatitidis* CYA. Each antigen sample was treated at 100°C for 10 min with 0.125 M Tris-HCl buffer (pH 6.8), 2% sodium dodecyl sulfate (SDS), 10% glycerol, 5% 2-mercaptoethanol and 0,025% bromophenol blue. Electrophoresis and immunoblot was conducted as previously described [22]. Membranes were blocked using 5% skin milk solution in TBS. After 3 washes with TBS, membranes were cut and probed separately by incubating with 5 µg/mL of each mAb.

### Yeast damage by oxidative stress condition

The sensitivity of yeast cells to oxidative stress was measured by two different assays.

- Halo assay. *H. capsulatum* yeast cells (10^5^) were plated on HAM-F12 agar plates with 5-mm-diameter filter paper discs (Whatman- Maidstone, England) embedded with different stock concentrations of H_2_O_2_ (0, 0.5. 1, 2, 3 M) at the center of the plates. Plates were incubated for 7 days at 37°C. The diameter of the clearance zone was measured by multiplying the distance from the embedded H_2_O_2_–containing disk to the nearest colony by two.
- Liquid cultures. Oxidative stress in liquid culture was evaluated by growing the cells with different concentrations of H_2_O_2_ (0, 0.5, 1, 1.2, 1.4, 1.6, 1.8, 2, 5, 10, 20 mM). Yeast cells were inoculated onto 50 mL of HAM's F-12 medium at an initial concentration of 10^6^ cells/mL and grown at 37°C with 150 rpm shaking. Growth was determined by hemocytometer counts and by colony forming units (CFU) by plating aliquots from the culture every 24 h on BHI agar, supplemented with sheep blood cells (50 mL per liter of medium) (Colorado Serum Company, Denver).

### Catalase activity

Two methods were used to assay catalase activity in *H. capsulatum*. First, yeasts were grown in HAM F-12 at 37°C and aliquots from culture were collected at different intervals. Catalase activity was measured as described in the Worthington enzyme manual (Worthington Biochemical Corp., Freehold, N.J., 1972)[45]. Decomposition of hydrogen peroxide was measured spectrophotometrically at 240 nm. Measurements were made at 20-s intervals for the first 3 min after the cells were mixed with the substrate. One unit of catalase was defined as the amount required to catalyze the decomposition of 1 mmol of hydrogen peroxide per min in 0.05 M hydrogen peroxide at 25°C.

The presence of the catalase activity in different cellular fractions of yeasts was also evaluated using the Catalase Assay kit (Cayman Chemical, Ann Arbor, MI, USA). Supernatants, intact cells, acetone permeabilized cells, sonicated cells, disrupted cells plus supernatant, soluble fractions after disruption and centrifugation, soluble fractions after disruption and centrifugation plus supernatants were used in this experiment. First, 10^6^ *H. capsulatum* yeast cells were inoculated in 100 mL of HAM's F-12 medium and aliquots of 1 mL of the culture obtained daily during the growth (24, 48, 72, 96, 120 and 144 h) and the number of yeasts determined. Supernatants were obtained after centrifugation of the aliquots at 1,100×*g* for 10 min. Equal number of cells from each day were permeabilized using acetone for 20 min at −20°C or disrupted using ultrasonication with 10 cycles of 1 min followed by incubations on ice. Cells were disrupted in the presence (characterizing total activity) or absence of supernatant and submitted to centrifugation at 11,300×*g* for 10 min to isolate the soluble cellular fractions. The fractions were suspended in 50 mM potassium phosphate pH 7.0 containing 1 mM EDTA. The assay was performed using formaldehyde as a standard for the catalase reactions and bovine liver catalase was used as a positive control. Assay buffer containing hydrogen peroxide was added to each well of the plate and the reactions incubated on a shaker at room temperature for 20 min. Reactions were stopped by adding potassium hydroxide and developed with Purpald (4-amino-3-hydrazino-5-mercapto-1,2,4-triazole, Cayman) which specifically forms a bicyclic heterocycle with formaldehyde and turns purple when oxidized. Plates were read at 540 nm. The catalase activity for each sample was calculated based on the standard curve obtained from formaldehyde samples and plots of catalase activity versus time were constructed for each cellular fraction evaluated.

### Localization of the M antigen

Cell wall/membrane extracts (CW/M) of strain G217B were produced as described [37]. Briefly, yeast cells were centrifuged at 1,100×*g* for 10 min and pellet washed three times with PBS. Yeasts were killed by treatment for 1 h at 25°C with thimerosal (1∶10,000) and suspended in PBS containing protease inhibitor cocktail (Complete Mini – Roche, Mannhein, Germany). Cell disruption in an ultrasonicator was conducted with 10 cycles of 1 min followed by 1 min incubations on ice and the efficacy of disruption was confirmed by microscopy (Figure S1A and B). The yeast homogenate was centrifuged at 1,100×*g* for 10 min. Pellet and supernatant were collected and the supernatant centrifuged at 11,000×*g* for 20 min at 4°C and isolated. The two pellets were pooled and washed three times using PBS. Then, the particulate material was boiled in 125 mM Tris pH 6.9, containing 6 M urea, 20 mM β-mercaptoethanol and 1% Tween 20 for 10 min. The samples were incubated at 4°C overnight to optimize the solubilization of the material. Supernatant was isolated after centrifugation and the soluble material dialyzed against PBS for 36 h to remove detergent. Immunoblots using 1 µg/mm of CW/M preparation and mAbs against M antigen were done as described above.

### Immunofluorescence

*H. capsulatum* yeast cells grown for 72 h at 37°C were centrifuged at 1,100×*g* for 10 min and the pellet washed three times with PBS. The concentration of cells was enumerated by hemocytometer and 5×10^6^ yeasts were suspended in 100 µl of a solution containing 10 µg/mL of a mAb against the rM diluted or an isotype control mAb (Mouse IgG2a-unlabeled, clone HOPC-1, Southern Biotechnology) in blocking buffer (BSA 1% in PBS). The cells were incubated for 1 h at 37°C, washed three times with PBS and the pellet suspended in 100 µl of anti-mouse IgG conjugated with FITC at a 1∶100 dilution in blocking buffer and incubated for 1 h at 37°C. After 3 washes, cells were suspended in 50 µl of a mounting solution containing 0.01 M of *N*-propyl galate diluted in PBS/glycerol (1∶1 vol/vol). Ten microliters of the suspension was applied to a microscopy slide and examined in a fluorescence microscope using a 495 nm filter, with a magnificence of 100×.

### Co-immunoprecipitation

Cell-wall extracts obtained as described above, except that no urea was present in the extraction buffer, were pre-incubated with the mAb 6F12 and an isotype control mAb (Mouse IgG2a-unlabeled, clone HOPC-1, Southern Biotechnology) overnight at 4°C. Protein A/G UltraLink resin slurry was added to the mixture (Pierce Biotechnology) and incubated for 2 h at room temperature. The agarose beads were pelleted by centrifugation at 2500×g for 5 min and supernatants used to measure the catalase activity by spectophotometric assay at 240 nm, as previously described [45]. Co-immunoprecipitates were additionally treated with glycine buffer (0.1 M, pH 2.5) and catalase activity evaluated, as well as immunoblots to detect the presence of the M antigen in this fractions.

### Catalase detection during cell growth

Yeast cells were grown in HAM F-12 medium and aliquots were taken from the culture daily for 14 days. Yeast cells were centrifuged at 1,100×*g* for 10 min and supernatants were collected. The pelleted yeast cells were washed three times with PBS then disrupted as described above. The yeast homogenates were centrifuged at 1,100×*g* for 10 min then the pellets and supernatants were collected. The supernatants were centrifuged at 11,000×*g* for 20 min at 4°C and isolated (cytoplasmic fraction). The pellets were pooled and used for cell wall/membrane extraction as described previously. All collected material was stored at −20°C prior to testing. The presence of M antigen in the supernatant was evaluated by immunoblot using a mAb to the rM protein. The viability of cells during growth was assessed by counting using propidium iodine, CFU plating and measurement of lactate dehydrogenase activity (LDH) in supernatant (concentrated 20×) and cytoplasmic fractions (Cytotoxity detection kit, Roche, Mannheim, Germany).

### Statistical analysis

All the statistical analysis was performed using GraphPad Prism version 5.00 for Windows (GraphPad Software, San Diego California USA). A one way ANOVA using a Kruskall-Wallis non-parametrical test was used to compare the differences between groups. A 95% confidence interval was considered in all experiments.

## Results

### Sequence analysis and phylogeny studies on the M antigen

**Figure 1:**
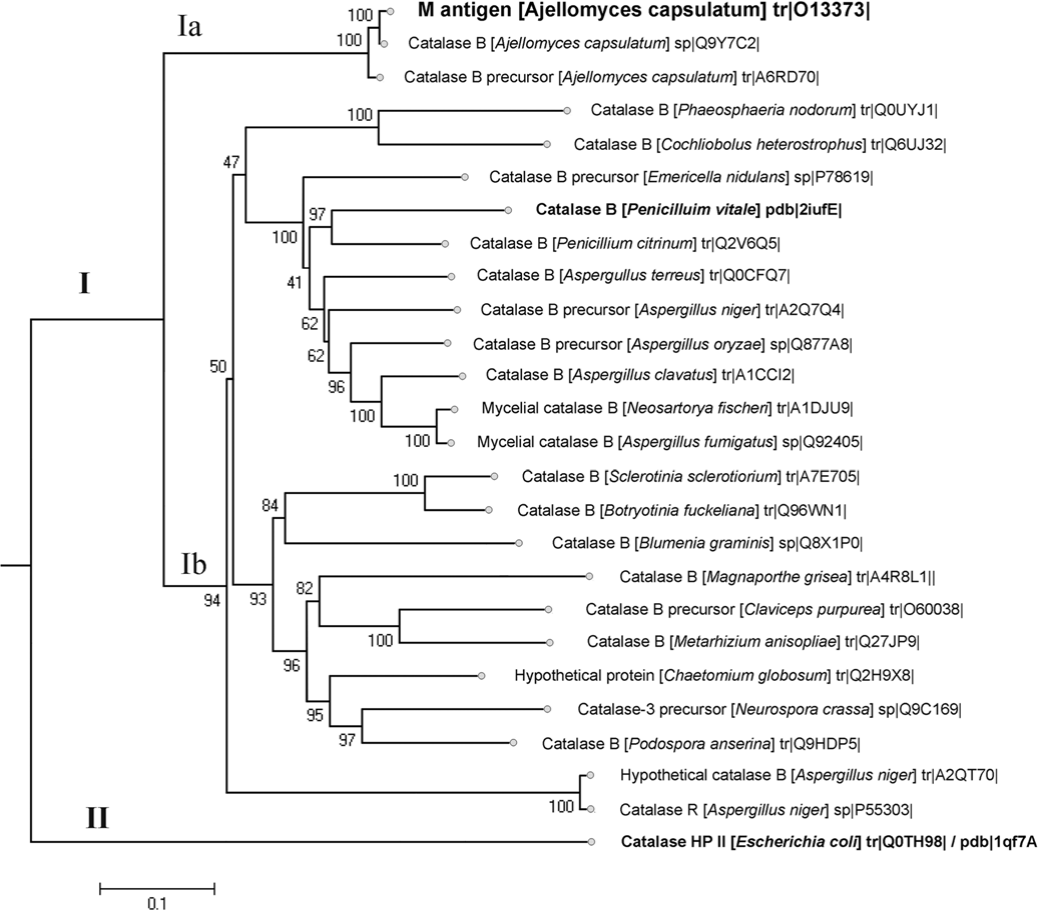
Phylogenetic analysis and dendrogram comparing the amino acid sequences of several catalase-peroxidases from the *Ascomycota* class and catalases used to construct the M antigen model (shown in bold). Swiss-Prot (sp) accession numbers for each sequence are shown on the right. Alignment of 26 catalase sequences was done using CLUSTAL W. The sequences were subjected to phylogenetic analysis using neighbour-join with maximum parsimony and minimum evolution, and length of the lines and distance between the clusters determined. The numbers on the branches are bootstrap values obtained with 500 replicates and indicate the frequency that all species to the right appear as a monophyletic cluster.

The encoded M antigen shared ∼80% identity with catalases from *Aspergillus* species, *Emericella nidulans* and other reference catalases from the *Ascomycota* using BLAST program (result not show). We conducted phylogenetic analysis of 25 sequences of the catalases B family from different fungal species and the *E. coli* catalase (HP II) used as sample in the modeling of the M antigen. Sequence analysis was performed on amino acid sequences deduced from nucleotide sequences. Phylogenetic analysis of sequences by neighbor-joining method (Figure 1) showed the distinct clustering of catalases. Catalases are divided into two clades (clade I and clade II), supported strongly by the bootstrap test (result not show). Clade I contains the M antigen (Clade Ia) and catalases from *Aspergillus* and *Penicillium* genera (Clade Ib) and also grouped catalases from other analyzed species. However, *E. coli* catalase (HP II) was in a difference clade (clade II) with a higher phylogenetic distance, as expected. There was 100% similarity between the M antigen (tr|013373|) and the catalase B (sp|Q9Y7C2|) sequence of *H. capsulatum*, which was expected as these sequences come from the same protein except the M antigen lacks the signal-peptide contained in catalase B sequences [15], [46].

### M antigen model resembles a catalase structure

**Figure 2:**
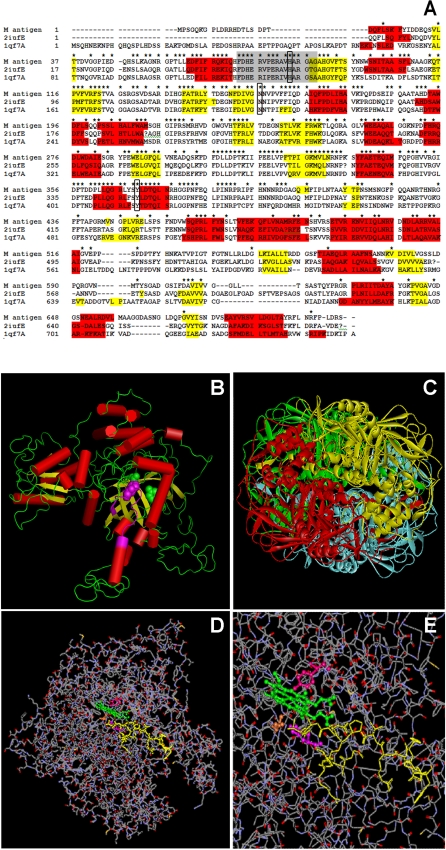
Studies of homology modeling of the M antigen. (A) Sequence alignment by CLUSTAL-W and comparison of catalases of *Histoplasma capsulatum* (M antigen), *Penicillium vitale* (2iufE) and *Escherichia coli* (1qf7A) used in the construction of the 3-D model of the M antigen. Amino acids are shown in single-letter code and stars indicate conserved amino acids residues and exact identity among the sequences. Residues highlighted in red and in yellow show α-helix and β-sheet secondary structures, respectively, determined by JPRED. Residues aligned in gray represent the catalase proximal active site signature. Boxes indicate the most favorable sites for heme ligand docking (H_83_, N_156_, Y_370_). Numbering of the residues and identifications for each protein are indicated to the left of the protein sequences. (B) Hypothetical model of the M antigen based in structure homology with other catalases. The proximal active site signature is shown in pink within the sequence. Cylinders represent alpha helixes and arrows, beta sheets. (C) Hypothetical model of the M antigen as a tetramer showing the *N*-terminal (blue), barrel shaped domain (red), binding domain (yellow) and helicoidal domain (green). (D) Structure showing the signature site of M antigen as a catalase and (E) Folded structure showing the insertion of the heme group (green) by docking, showing the respective interaction sites, H_83_ (magenta), N_156_ (orange), Y_370_ (pink).

Using homology modeling, a three-dimensional model was constructed for the M antigen based on the structures of the *P. vitale* (PDB ID 2IUF) and *E. coli* (PDB ID 1QF7) catalases. The final sequence alignment between M antigen, *P. vitale* and *E. coli* catalases is shown in Figure 2A. The optimal alignment contained eight insertions and one deletion of the M antigen predicted structure. The structure of M antigen resembled that of a catalase (Figure 2B). The deletion at position 599 to 613 in relation of *P. vitale* catalase and insertion at position 660 to 668 in relation to the M antigen represents the greatest difference between the M antigen and *P. vitale* catalase. The other insertions can be considered as non-breaking gaps. In its hypothetical form, the M antigen is arranged in solution as dimers or tetramers (shown in Figure 2C). The model allowed for the identification of possible binding sites of catalase by comparison with other reference catalase enzymes, suggesting that their biological function should be similar. When the sequence was analyzed for common residues, we found a consensus catalase sequence, consisting of (_72_FDHERVPERAVHARGAG_88_) as shown in Figure 2C. From the structure-structure comparison of the template, it was found that secondary structures were highly conserved. The active site formed a cleft (Figure 2D) and within the sequence, a consensus pattern consisting of R - [LIVMFSTAN] - F - [GASTNP] - Y - X - D - [AST] - [QEH] was identified with conserved residues such as histidine (H83), asparagine (N156) and isoleucine (Y370) that was the most favorable site to dock the heme ligand and permitted the design of a possible site of insertion of this group (Figure 2E).

### Epitope mapping of the rM antigen

**Figure 3:**
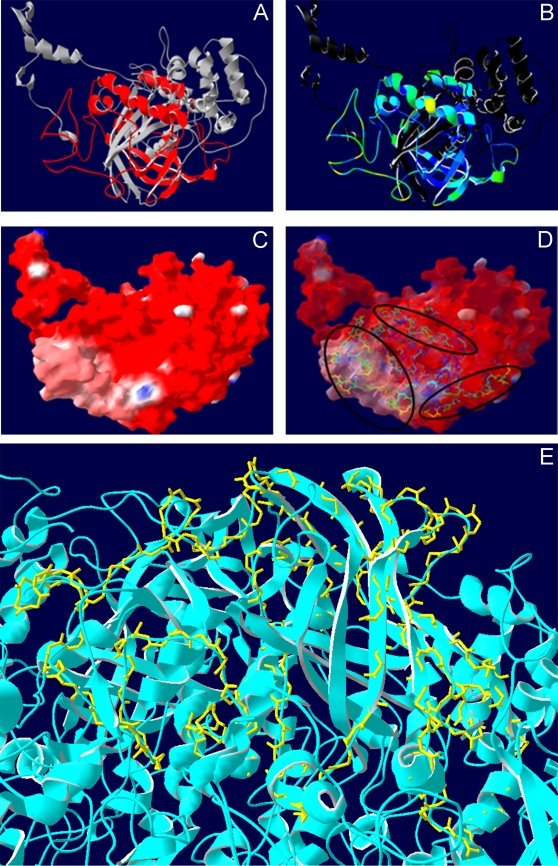
Topology studies of fragment 2. (A) M antigen ribbon representation of the molecular model, with F2 colored in red. (B) F2 structure colored for solvent accessibility where blue indicates least accessibility. (C) Mapping of the surface of the M antigen. The surface has been made transparent to allow the perception of the internal protein architecture and colored according to the Coulombic electrostatic potential: red, negative; grey, neutral; and blue, positive. (D) Top view of the F2 on the surface of the M antigen model with ribbons colored by accessibility (from blue to red indicating less to more, respectively). Circles indicate the regions with highest antigenic index and accessibility. (E) Top view of F2 (backbone-yellow) with the ribbon molecular model of the tetramer form of the M antigen. The secondary structure, solvent accessibility and molecular surfaces were calculated in SwissPDB Viewer v3.7.

Using Jamenson-Wolf algorithm (Protean program, DNASTAR Inc, Madison, Wis., USA), we predicted antigenic regions in the complete protein (result not shown) and determined that the region between amino acids 212 to 442 displayed the highest number of predicted epitopes. We further characterized the localization, secondary structure, solvent accessibility and molecular surfaces of F2 in the constructed structure of the M antigen with the SwissPDB Viewer v3.7 software (Figure 3). F2 was present at the surface of the molecule (Figure 3A) and had a very high accessibility to solvents (Figure 3B). When the surface of the structure was mapped and colored according to electrostatic potential, most of F2 was exposed, displaying a neutral charge (Figure 3C) and readily accessible to antibodies or host effector cells (Figure 3D). In the tetramer structure of the M antigen (Figure 3E), F2 remained exposed on the surface.

Based on this analysis, we decided to clone this fragment, called fragment 2 (F2), as well as the sequence before fragment 2, called fragment 1 (F1), and the sequence after, fragment 3 (F3). The sum of the area for all the epitopes identified (i.e., the sum of antigenic index of each residue) in each fragment by the DNAstar was compared with the results of specificity of Ig detection in sera from patients with histoplasmosis. F2 showed an area under curve (AUC) 28.8% higher than F1 or F3 (p<0.001), and there was no statistical difference between the AUCs of F1 and F3. Table 2 shows the comparison among the fragments and the immunodominant score of F2.

**Table 2:** Comparison of the antigenic index of the fragments generated from the recombinant M antigen.

	Total antigenic index^a^	AUC^b^	Antigenic index average^c^ (Total /number of residues)
F1 (194aa)	123.66	134.34	0.692
F2 (231aa)	**207.15**	**205.95**	**0.892**
F3 (263aa)	178.45	183.50	0.698

AUC means the integral of the area and the sum of residues score.

a Absolute values corresponding to the sum of each residue in the sequence.

b Integral of the curve representing the antigenic index and calculated area.

c Average of the antigenic index values calculated by dividing the total antigenic index by the number of residues of the considered fragment.

**Figure 4:**
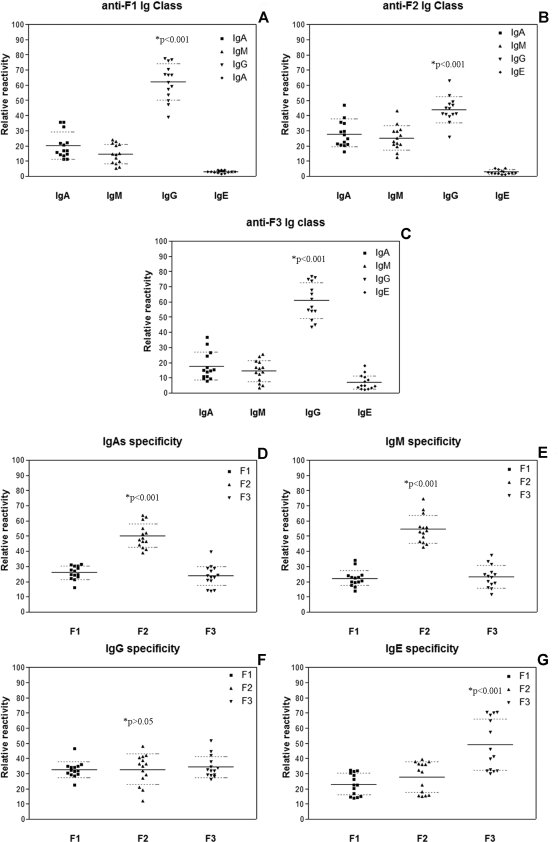
Pattern of immunoreactivity of the M antigen fragments and determination of antibodies specificity in sera of patients with histoplasmosis. Figure shows the distribution of patients' sera tested for each immunoglobulin class/fragment. Each symbol represents a single individual's serum. Dashed lines (……) represent the error bars and indicate a 95% confidence level; *P* values were determined using the One-way ANOVA test.

Reactivity of sera from patients with histoplasmosis against rM and rM fragments were evaluated for the presence of IgA, IgM, IgG and IgE and the reactivity of each fragment was compared. Intragroup reactivity was defined as the reactivity of each class of Ig for each fragment specifically, and intergroup reactivity was the reactivity of a single class of Ig to each fragment. The relative reactivity inter/intragroup (percentage of total reactivity) determined in each case is shown in Figure 4. For each fragment, IgGs were the most reactive, followed by IgAs, IgMs and IgEs (*p*<0.001). When antibody classes against F1 and F3 were assessed, the IgGs recognized epitopes on F2 better than Igs from the other groups (*p*<0.001) (Figure 4A and C). For F2, the IgGs were the most reactive (*p*<0.001), but IgAs and IgMs also accounted for a greater percentage of reactivity compared to the results from F1 and F3 (Figure 4B). This was further clarified when the specificities of each class of Ig were compared for each fragment where IgAs and IgMs were shown to have the most reactivity to epitopes on F2 (*p*<0.001) (Figures 4D and E, respectively). In contrast, IgGs reacted equally against the three fragments tested and no statistical difference was observed (*p*>0.05) (Figure 4F). Although IgEs contributed the least to overall reactivity, most reactivity occurred to F3 (*p*<0.001) (Figure 4G). These results support the hypothesis that different classes of Ig recognize different epitopes in distinct regions of the antigen and that the Igs have different specificities. Comparing data from ELISAs with the results of epitope mapping and prediction using bioinformatics, the fragment ELISA for epitope mapping and the computed antigenic indices show that F2 was the most immunogenic segment of M antigen.

### mAbs against rM protein

The three mAbs to rM antigen with the highest reactivity by ELISA (IgM mAb 7C7 and IgG2a mAbs 6F12 and 8H2, data not shown) were used to probe antigenic preparations from microorganisms that historically cross react in serologic tests with *H. capsulatum*. The reactivity profiles of mAbs were determined according to the bands seen after developing immunoblots and calculations of the respective molecular weights (Table 3, Figure S2). The mAb 8H2 showed the highest specificity, since it did not recognize any protein in the antigenic extracts from *P. brasiliensis*, *A. fumigatus*, *S. schenckii*, *B. dermatitidis* or *M. tuberculosis*. MAbs 7C7 and 6F12 recognized multiple bands in the molecular weight range of 60 to 80 KDa from filtered antigens of mycelial *P. brasiliensis*. These mAbs also reacted nonspecifically against CYA antigen from *P. brasiliensis*. None of the mAbs tested reacted against antigenic preparations from *A. fumigatus*. MAb 6F12 showed reactivity with a 60 KDa and a 17 KDa protein from *S. schenckii* extract. Multiple bands could be the result of protein degradation and/or that the epitope recognized by the mAbs are shared by different antigens. In the case of the immunoblots with the rM antigen, the multiple bands observed are due to protein degradation during the purification process. Hence, mAb 8H2 appears to be specific for the M antigen of *H. capsulatum* and may have potential use in the diagnosis of histoplasmosis.

**Table 3:** Pattern of recognition of proteins by the monoclonal antibodies used in specificity studies by immunoblot.

Antigen	Monoclonal Antibodies		
	8H2	7C7	6F12
Ag rM	**100**,80,60,50,40,35,30,25-24,22-20 kDa	**100**,80,35,25,24 kDa	**100**,80,60,50,40,35,30,25-24,22-20 kDa
pHMIN	X	100,**80** kDa	X
Hc CYA	X	**80**, 50 kDa	100,**90**,70,61,25 kDa
Pb339 CYA	X	90,80,70,61,52-50,20 kDa	61,55,50,40,20 kDa
Pb(f)	X	80-60 kDa	X
Af (son)	X	X	X
Af (f)	X	X	X
Bd CYA	X	50 kDa	X
Ss CYA	X	X	60, 17 kDa
Mt (son)	X	X	X

Pb339 CYA - cytoplasmic yeast antigen of *Paracoccidioides brasiliensis*; Pb (f) - filtrated antigen of *P. brasiliensis*; Af (son) – sonicated antigen of *Aspergillus fumigatus*; Af (f) - filtrated antigen of *A. fumigatus*; Bd CYA - cytoplasmic yeast antigen of *Blastomyces dermatitidis*. Ss CYA - cytoplasmic yeast antigen of *Sporothrix schenckii*; Mt (son) - sonicated antigen of *Mycobacterium tuberculosis*. Ag rM - recombinant M antigen of *Histoplasma capsulatum*; CYA Hc - cytoplasmic yeast antigen of *H. capsulatum*; pHMIN - purified and periodate treated histoplasmin. The numbers listed show the molecular weight of the protein recognized when probed with each monoclonal antibody. Absence of reactivity is indicated by an ‘X’.

### Fungal susceptibility to oxidative stress by hydrogen peroxide

**Figure 5:**
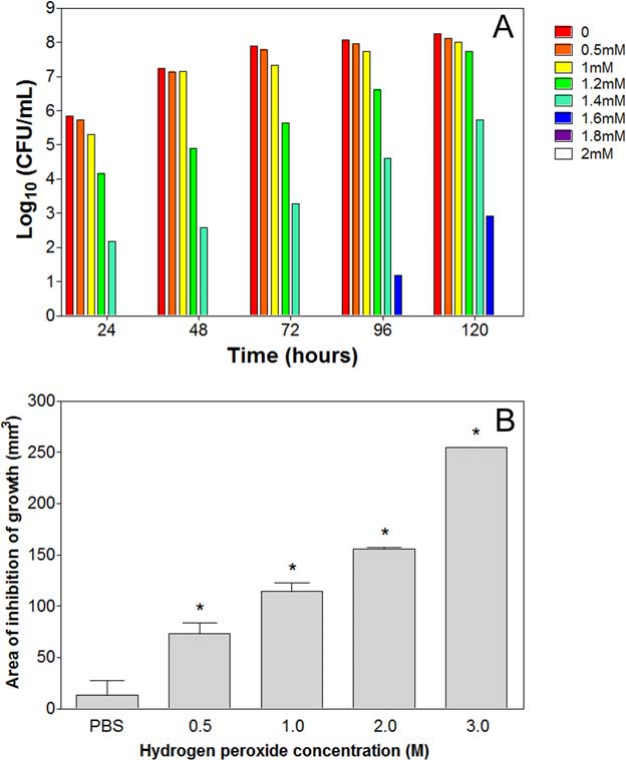
Effects of oxidative stress by hydrogen peroxide to *H. capsulatum*. (A) Susceptibility of yeasts to different H_2_O_2_ concentrations during growth of yeasts in HAM F-12 liquid medium by measurement of cell viability by CFU determinations, with no difference among concentrations up to 1 mM (p<0.05) at each time interval. Results show the average of three independent experiments. (B) Halo assay comparing the hydrogen peroxide sensitivity of strain G217B. Liquid culture was grown to mid log phase and plated on HAM-F12 agar plates to form a yeasts lawn. A filter disc containing the indicated concentration of hydrogen peroxide was placed at the center of each plate. After incubation for 7 days, the clear zone surrounding each filter disc was measured (inhibition of growth) and plotted versus concentration of H_2_O_2_. Increasing concentrations of H_2_O_2_ show more inhibition of growth (p<0.001). Each experiment was performed in triplicate and repeated at least three times.

To determine the role of catalases to *H. capsulatum* sensitivity to oxidative stress *in vitro*, strain G127B was grown in different concentrations of H_2_O_2_ (0, 0.5, 1, 1.2, 1.4, 1.6, 1.8, 2, 5, 10, 20 mM). There was no statistical difference in the rate of growth of *H. capsulatum* yeasts as enumerated by hemacytometer with concentrations ranging from 0 to 1.0 mM of hydrogen peroxide (*p*>0.05), whereas progressive reduction in growth was observed with increased concentrations of hydrogen peroxide up to 1.6 mM and no growth occurred by 120 h at concentrations ≥1.6 mM (*p*<0.001). CFU determinations revealed that no viable cells were recovered from cultures with concentrations ≥1.8 mM of hydrogen peroxide (Figure 5A). As described previously [47], H_2_O_2_ challenge in liquid medium represents a short duration exposure for catalase expressed by microorganisms (since H_2_O_2_ is intrinsically unstable). To test the effect of a more prolonged exposure, the halo assay was performed. Figure 5B shows the halo diameter of the yeast after placing the filter discs with H_2_O_2_ concentrations ranging from 0 to 3 M. As expected, more killing occurred with increased oxidative stress and, consequently, a bigger halo was formed compared to the PBS control (*p*<0.001).

### Catalase activity

**Figure 6:**
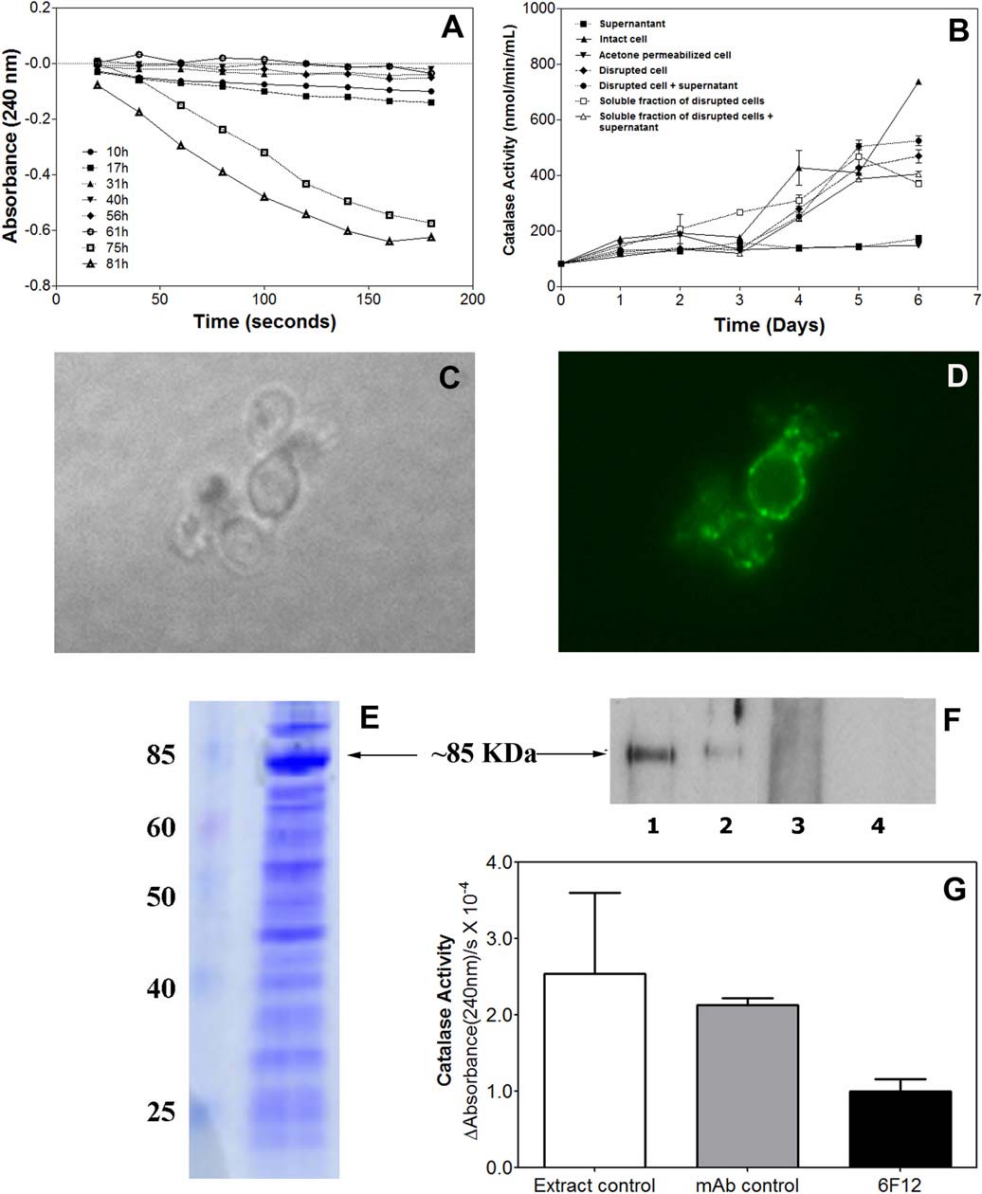
Catalase activity in G217B yeast cells. (A) Measurement of catalase activity during growth of yeasts by measuring the decomposition of H_2_O_2_ at 240 nm. Decreased absorbance corresponds to breakdown of hydrogen peroxide due to catalase activity. (B) Catalase activity in different cell fractions and its variation over time. Error bars indicate confidence level (95%); *P* values were determined using One-way ANOVA test. (p<0.001). Each sample was tested in duplicate and the experiment was repeated three times. (C) Light and (D) immunofluorescence microscopy of yeast cells showing the immunolocalization of M antigen on the surface of *H. capsulatum* using the 8H2 mAb. Similar reactivity occurred with mAb 6F12 and 7C7. (E) SDS-PAGE of the cell wall/membrane yeast extract preparation. (F) Immunoblot showing the reactivity of mAbs against the rM antigen to cell wall/membrane yeast extract. Lines 1, 2 and 3– mAbs 8H2, 6F12 and 7C7 against the M antigen, Line 4 – Irrelevant immunoglobulin as a negative control. (G) Co-immunoprecipitation of M antigen from the cell wall/membrane extracts using mAb 6F12 showing a reduction of the catalase activity of this fraction comparing to the PBS and isotype control.

We measured the catalase activity in the supernatant of *H. capsulatum* yeast cultures by exploiting the absorption spectra of hydrogen peroxide to provide an indirect method of observation and quantification of catalase activity. Absorption of UV light (240 nm) corresponds to levels of hydrogen peroxide present in the sample. Aliquots from the culture at early stages of growth (0 to 61 hrs) had undetectable catalase activity, whereas degradation of hydrogen peroxide was observed in aliquots from cultures older than 61 hours (Figure 6A). When the catalase activity was normalized by the number of cells, no difference was observed (data not shown).

Different cellular fractions were obtained by sonication/ centrifugation protocols from the *H. capsulatum* yeast grown in liquid medium and the catalase activity of the materials sequentially obtained were measured (Figure 6B). Catalase activity was not detectable in supernatants with the system used. Intact cells were used to confirm the hypothesis that catalase was not been secreted and it could be localized to the cell wall and the cells showed higher catalase activity after 6 days of growth. Cytoplasmic extracts obtained by cellular permeabilization showed an absence of intracellular enzyme activity. The catalase activity in the disrupted cell fraction was lower than to the intact cell group, suggesting that the main location of activity resides on the *H. capsulatum* yeast cell surface. Notably, a reduction in activity was observed in the soluble fractions of the yeast cells when compared to disrupted cells (∼100 nmol/min/mL), indicating that the catalases are distributed in soluble and insoluble fractions, which are precipitated at high speeds, possibly on fragments of cell wall. These results support the hypothesis that the catalases are located where they can protect the cell wall against oxidative stress.

### M antigen localization on yeast cells of *H. capsulatum*

The results obtained with localization of catalase activity led us to further study the location of the M antigen on yeast cells by immunofluorescence and immunoblot using the highly immunoreactive purified mAbs. The mAbs brightly reacted with *H. capsulatum* yeast by immunofluorescence of both mother and daughter cells (Figure 6C and D). Since the yeast was not permeabilized, this result suggests that the M antigen was in fact accessible at the cell surface. No reaction was observed using an irrelevant immunoglobulin as a negative control and mAbs against M antigen did not label yeasts of *P. brasiliensis* (Figure S3A and B). Furthermore, mAbs 8H12, 6F12 and 7C7 recognized a protein of ∼85 KDa on the immunoblot, consistent with the molecular weight of the M antigen (Figure 6E and F).

Co-immunoprecipitation of cell wall extracts with the specific mAb 6F12 against the M antigen showed a reduction of the catalase activity in this cell fraction. This mAb was used due to the best binding to the M antigen in its native conformation, as determined by ELISA (Figure S4A). After immunoprecipitating the M antigen in this fraction, a reduction of 60% and 53% in the catalase activity was observed compared to the PBS and mAb isotype control groups (p<0.001, Figure 6G). The co-immunoprecipitates of each condition were also evaluated for catalase activity and for the presence of the M antigen by immunoblot. The catalse activity in the mAb 6F12 co-immunoprecipitates were five times higher than controls and a more intense band was observed in immunoblots from the mAb 6F12 co-immunoprecipitates (Figure S4C and D, respectively). These data suggest that this protein is principally located at the cell wall where it may participate in protecting the fungus from oxidative stresses.

**Figure 7:**
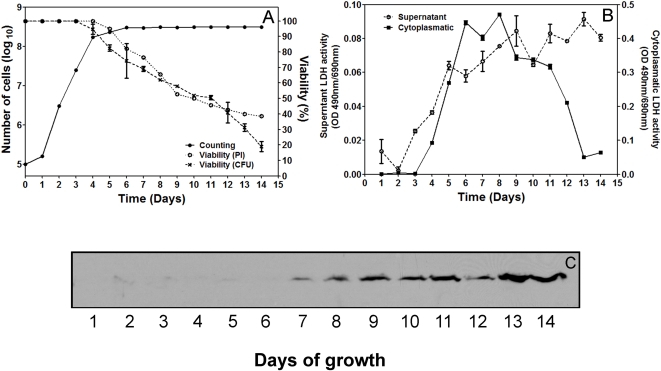
Detection of M antigen during growth of yeast cells. (A) Growth of *Histoplasma capsulatum* in HAMF-12 medium enumerated by hemocytometer and viability of G217B yeast cells determined by propidium iodine and CFU plating. (B) LDH activity in supernatant of culture and cytoplasm fractions of yeast cells during growth in the same conditions. Results show the average of three independent experiments. (C) Detection of M antigen in the supernatant during growth in culture by immunoblot using mAb 8H2.

When *H. capsulatum* was grown in HAM F-12 medium for two weeks, the M antigen was detected in the supernatant when the yeast cells viability was lower than 70% (Figure 7A) and LDH activity concomitantly increased, consistent with cell death (Figure 7B). This suggests that the M antigen released from the yeast cells to the supernatant starting at day 7 (Figure 7C) arises from yeast cell lysis with release of cytoplasmic contents, and not from secretion into the supernatant as exoantigen as described previously for the filamentous phase of the fungus [48], [49].

## Discussion

The M antigen of *H. capsulatum* has been characterized as a catalase (CatB) by molecular methods [15], yet little is known about its biological function. In *H. capsulatum*, there are three identified catalases that are non-related, but all have a heme group in their theoretical structures and show a conserved core (N-terminal, barrel shaped domain, binding domain and helical domain) of 300 amino acids. The highest similarity of the M antigen to other catalases is found in the region surrounding the distal and proximal tyrosine and the catalase signature site [7], [50]. The molecular model of the M antigen resembles the structures and domains of a catalase, including the heme binding site, such as the ligands H_107_, N_180_ and Y_394_ (Figure 2C and D).

To study the importance of *H. capsulatum* catalases, we evaluated fungal susceptibility to oxidative stress by hydrogen peroxide. The physiological concentration of H_2_O_2_ ranges 30–50 µM [51], [52]. We found that the growth and morphology of *H. capsulatum* was not affected by exposure to 1 mM H_2_O_2_, twenty-times higher than physiological concentration. *H. capsulatum* defense against damage by H_2_O_2_ could be achieved using catalases by either of two strategies: catalases could be localized on the cell wall providing local protection and/or could be secreted into the extracellular milieu to detoxify the local growth environment.

Catalases have been detected in cell-free extracts or in solution after permeabilizing yeasts of *H. capsulatum*, suggesting that these were secreted enzymes [53]. We evaluated several cellular fractions from *H. capsulatum* yeast cells for catalase activity. The highest activity was seen in intact cells and the activity of disrupted cells plus collected cytoplasmic extracts were statistically similar (p<0.05). This data suggests that the enzyme was not significantly secreted in the conditions used and/or that it accumulated on the cell wall. Interestingly, we recently detected by mass spectrographic methods M antigen in extracellular vesicles of *H. capsulatum* isolated by ultracentrifugation from 2 day old cultures [54]. Hence, there may be some secretion of M antigen, but it is below the level of detection using the assays used in the current work (limit of detection of catalase activity by our microplate test is 0.0155 µg/ml (data not shown). Furthermore, it is likely that the M antigen is transported in vesicles to the cell surface and the isolated vesicles had not yet been trafficked to their appropriate location and deposited their cargo. In solid media, catalase activity could not be detected in the agar surrounding colonies of *H. capsulatum*, suggesting that this enzyme is not constitutively secreted (data not shown). Our results from the detection of the M antigen by immunoblot using mAb 8H2 and a cell wall/membrane extract confirmed the presence of the antigen in this fraction.

Although the M antigen had been characterized as an exoantigen in the mycelial phase, our results indicate that M antigen is not secreted in the yeast phase. We cannot discard the possibility that previous studies on mould forms of the fungus were either detecting catalase in the supernatant arising from dying/dead cells in the stationary phase in culture or that the mould forms have mechanisms for the extracellular secretion of this protein. Additionally, acetone treatment previously used during isolations could have extracted this protein from the cell wall. In the yeast stage, catalase activity in supernatant and the M antigen could be detected only after seven days, when *H. capsulatum* cultures display a high cell death rate. Hence, detection of the M antigen in supernatant is based on liberation of the protein after cell lysis.

Catalases are involved in detoxification and in virulence, and they have been reported to be located mainly on cell surfaces [55], [56]. During *H. capsulatum* yeast phase growth, the M antigen (catB) and catalase P (catP) are constitutively expressed, whereas catalase A (catA) is poorly expressed [5], [53]. The catP has been described to traffic to peroxisomes, were they can facilitate detoxification of hydrogen peroxide generated during metabolism [5]. *P. brasiliensis*, catP was similarly localized within cytoplasmic yeast extracts [10]. Using mAbs to the *H. capsulatum* M antigen, we determined that the protein is located on the yeast cell surface, presumably making the M antigen the most important catalase for detoxification of host derived peroxides. We cannot discard the importance of the *H. capsulatum* catP, since its definitive cytoplasmatic localization is yet to be proven. In addition, previous results from our group showed the presence of the M antigen in the cell wall of mycelial phase of the fungus [57] suggesting that this protein is on the surface of the infectious form where it might be involved in evading oxidative stress, permitting the conversion of the fungus from mycelia to the parasitic yeast. The strong reaction of the mAbs to rM protein with the surface of the yeast form of *H. capsulatum* reinforces this hypothesis and corroborates the data previously described indicating that the catB gene is expressed in both forms of *H. capsulatum* [5]. Hence, we surmise that the catalase activity detected on the surface is due to the M antigen, though this needs to be confirmed in future experiments using gene deletion or RNAi methods.

The catalase family of proteins is an important target for humoral immune responses in several mycoses, including during *A. fumigatus* infection [58], due to its localization on the cell surface. Since the M antigen induces the first antibodies detectable by standard laboratory methodologies during the host immune response to infection with *H. capsulatum* [59], [60], we undertook the mapping of the epitopes on the protein in order to define the highest antigenic residues with the intent of gaining insights into its functional roles to facilitate the future development of more specific and sensitive diagnostic methods and for vaccine studies. We first computationally mapped the putative epitopes of the M using the Jameson-wolf algorithm [61] and predicted the region with the highest antigenic index. Subsequently three different fragments from the M antigen structure were generated and the binding of a series of mAbs produced using the whole protein were mapped. Sera from human patients with histoplasmosis were also used to assess the immunodominance of the three fragments, revealing that F2 was the most immunogenic region due to the presence of higher titers of antibodies from different classes of Igs against this fragment. Furthermore, we showed by modeling the M antigen that F2 contains the major immunodominant epitopes of the protein and that the fragment is located on the surface of the M antigen (Figure 7). Techniques using this fragment for antibody detection may be useful for clinical applications, since the hyperimmune patient sera showed high levels of antibodies against this fragment.

MAbs 8H2, 6F12 and 7C7 were tested against different preparations of other fungal antigens and reacted distinctly, suggesting binding to unique epitopes. Notably, mAb 8H2 had the highest specificity and recognized epitopes on the recombinant M protein that were not present on the other antigens evaluated. The M antigen is highly homologous with catalases of *Aspergillus spp.* [15], but mAb 8H2 did not bind any proteins in the filtrate or sonicated preparations from this fungus, suggesting that mAb 8H2 is a species-specific Ab. We postulate that the M antigen is released from dead/dying cell during infection by *H. capsulatum*, which will enable techniques using these mAbs to detect this immunodominant protein during infection. This approach could be particularly clinically useful in patients with impaired antibody production, such as individuals with HIV infection, where immunodiffusion techniques are inadequate [1], [62]. Additionally, given the surface location of the M antigen, these mAbs may be therapeutically effective in modifying the pathogenesis of histoplasmosis [63]. Future studies will be needed to establish the utility of these new reagents for diagnostic and therapeutic purposes.

## Supporting Information

Figure S1 Intact *Histoplasma capsulatum* yeast cells (A) are not labeled by propidium iodine (B). The protocol applied to the yeasts to obtain cell wall/membrane extracts results in the complete disruption of the yeast cells (C) and the resulting debris are labeled by propidium iodine (D).
(3.69 MB TIF)

Figure S2 Specificity studies of mAbs generated to rM antigen. This data corresponds to Table 3. (A) Pb339 CYA - cytoplasmic yeast antigen of *Paracoccidioides brasiliensis*; Pb (f)- filtrated antigen of *P. brasiliensis*; (B) Af (son) - cytoplasmic antigen of *Aspergillus fumigatus*; Af (f)- filtrated antigen of *A. fumigatus*; (C) Ss - cytoplasmic yeast antigen of *Sporothrix schenckii*; Bd- cytoplasmic yeast antigen of *Blastomices dermatitidis*; (D) Mt- cytoplasmic antigen of Mycobacterium tuberculosis; rM Ag -recombinant M antigen of *Histoplasma capsulatum*; (E) pHMIN- purified and periodate treated histoplasmin; CYA Hc- cytoplasmic yeast antigen of *H. capsulatum*.
(3.88 MB TIF)

Figure S3 Light and immunofluorescence microscopy of *H. capsulatum* yeast cells (A and B) showing the absence of reactivity using an isotype mAb control. *P. brasiliensis* yeast cells (C) were not labeled by mAb 6F12 (D). Similarly, mAbs 8H2 and 7C7 did not label *P. brasiliensis* (not shown).
(1.89 MB TIF)

Figure S4 (A) Indirect ELISA [ELISA wells coated with rM protein] showing the reactivity of the mAbs to the M antigen, irrelevant control mAb and hyperimmune mouse serum. (B) Immunoblot using mAb 6F12 assessing the presence of the M antigen in culture supernatant and different cellular fractions: 1- culture supernatant, 2- whole cell extract, 3- cytoplasmic extract, 4- cell wall/membrane extract, and 5- soluble fraction of cell wall extract. (C) Co-immunoprecipitates from the cell wall/membrane extracts using mAb 6F12 showing an increase of the catalase activity compared to PBS and isotype control. (D) Immunoblot showing the detection of the M antigen in co-immunoprecipitates from cell wall/membrane extracts using a mAb against the rM (7C7). Co-immunoprecipitates of 1- PBS (No beads), 2- Isotype control, 3- mAb 6F12 and 4- Agarose beads (no antigen).
(2.91 MB TIF)
