# Supplementary figures and images for: Biological Function and Molecular Mapping of M Antigen in Yeast Phase of Histoplasma capsulatum
Source: PLoS One. 2008 Oct 17;3(10):e3449. doi: 10.1371/journal.pone.0003449 (PMC2566600; doi:10.1371/journal.pone.0003449)

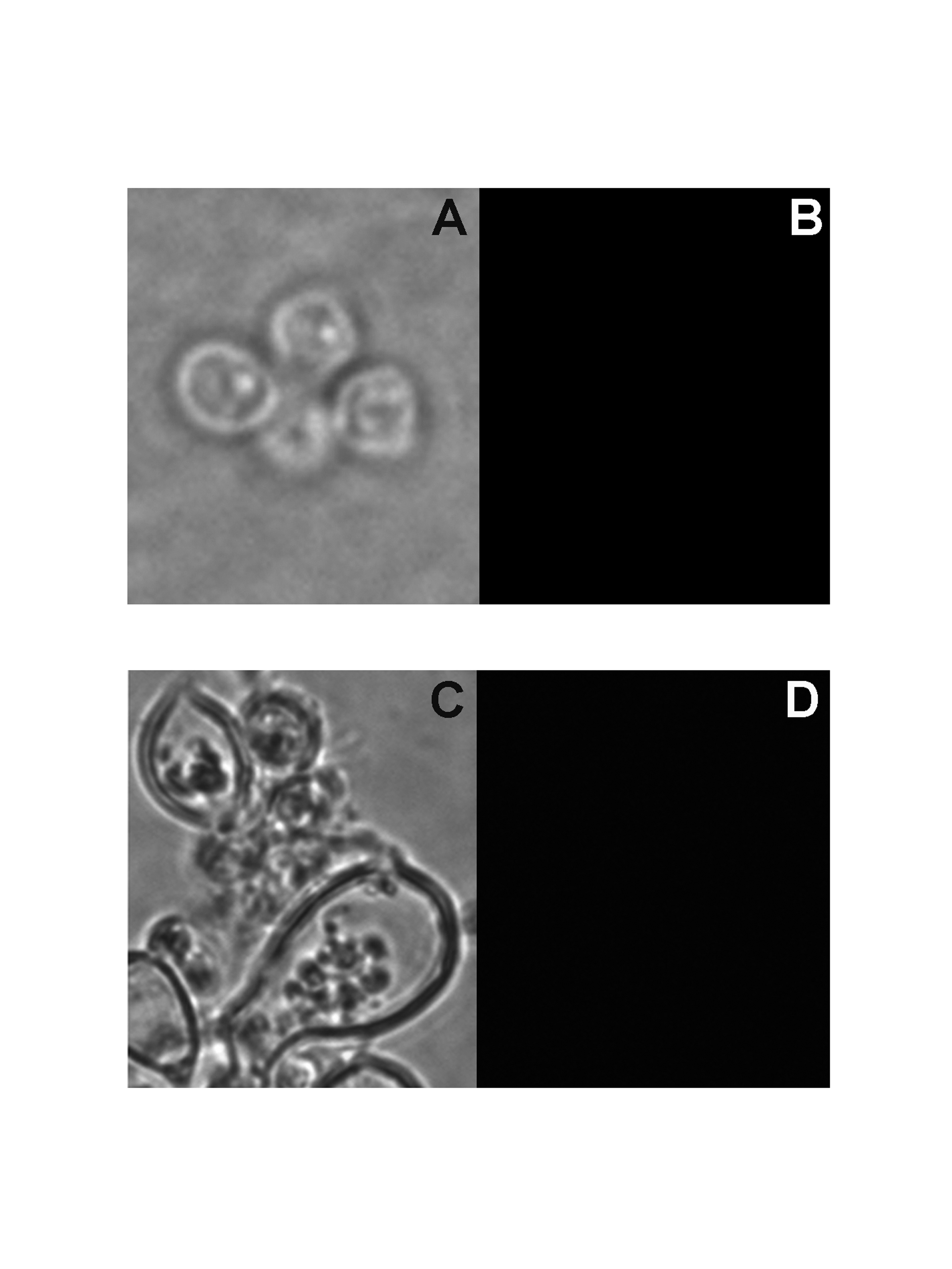

Supplement: Figure S3 — Light and immunofluorescence microscopy of H. capsulatum yeast cells (A and B) showing the absence of reactivity using an isotype mAb control. P. brasiliensis yeast cells (C) were not labeled by mAb 6F12 (D). Similarly, mAbs 8H2 and 7C7 did not label P. brasiliensis (not shown). (1.89 MB TIF) [file pone.0003449.s003.tif]
